# Optimized TELIP, an Echogenic Liposomal Nano-Carrier Loaded with Alteplase for Preclinical Studies

**DOI:** 10.3390/pharmaceutics18060646

**Published:** 2026-05-24

**Authors:** Maryam Ranjpour, Brion Frierson, Rebekah Lynn Emerine, Christian Jordan De Vera, Krishna Sarva, Melvin Earl Klegerman, David Dugald McPherson, Steven Idell, Galina Florova, Andrey Anatolievich Komissarov

**Affiliations:** 1 Department of Cellular and Molecular Biology, The University of Texas Health Science Center at Tyler, Tyler, TX 75708, USA; ranjpour77@gmail.com (M.R.); re9909@uttyler.edu (R.L.E.); christianjordan.devera@uttyler.edu (C.J.D.V.); krishna.sarva@uttyler.edu (K.S.); sidell@uttyler.edu (S.I.); galina.florova@uttyler.edu (G.F.); 2 Department of Internal Medicine, University of Texas Health Science Center at Houston, Houston, TX 77030, USA; brion.frierson@uth.tmc.edu (B.F.); melvin.e.klegerman@uth.tmc.edu (M.E.K.); david.d.mcpherson@uth.tmc.edu (D.D.M.)

**Keywords:** liposome, carrier, alteplase, sctPA, fibrinolysis, echogenic, targeted, delivery, encapsulation, TELIP

## Abstract

**Background:** Pharmacological treatment under conditions of slow fibrinolysis/thrombolysis requires the targeted delivery of plasminogen-activating activity. Echogenic liposomal formulations (regular TELIP) of single-chain tissue plasminogen activator (sctPA), while possessing high affinity to fibrin, contain free/loosely bound sctPA. We hypothesized that removal of free sctPA, which competes with liposomes and plasmin for fibrin, enhances unique features of the TELIP. **Methods:** Optimized and regular TELIP were assessed for the distribution of active sctPA (loosely bound, tightly bound, encapsulated), stability, binding to fibrin, initiating fibrinolysis in vitro and ex vivo using a battery of biochemical methods. **Results:** One milligram of the regular TELIP consists of 2.0–5.0 × 10^9^ echogenic liposomes (700–900 nm diameter). Non-specifically bound sctPA readily dissociates at the physiological ionic strength and pH. While up to 60% of sctPA in the regular TELIP is loosely bound with 6–15% encapsulated, and the rest is tightly bound to the liposomes; in the optimized TELIP, more than 80% of active sctPA is tightly bound with up to 40% of encapsulated. The latter is protected from high-molecular-weight ligands and could be released by an ultrasound pulse. Optimized TELIP shows low competition with plasmin for fibrin and effectively supports fibrinolysis in vitro and ex vivo. The optimized TELIP with maximal load of sctPA 3% (*w*/*w*) retains integrity at 37 °C for 5 h in vitro and up to 2 h ex vivo. **Conclusions:** The optimized TELIP is stable in vitro and ex vivo, does not interfere with fibrinolysis and retains a high level of encapsulated sctPA delivered precisely to the thrombus/fibrin clot.

## 1. Introduction

**Scheme 1:**
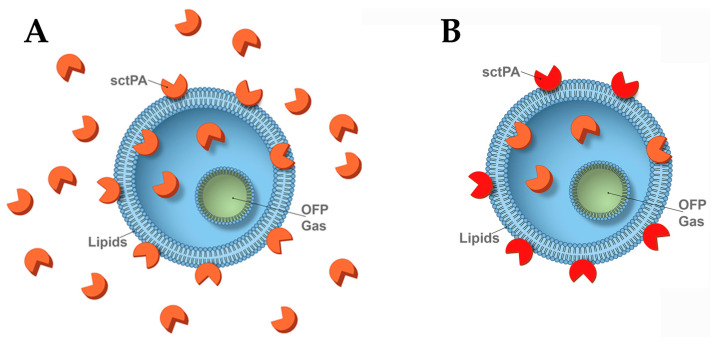
Representation of a single liposome of regular (**A**) and optimized (**B**) TELIP. Three types of active sctPA (orange) in TELIP are shown: (i) Loosely bound sctPA (up to 60% of active enzyme in the regular and 0–10% in the optimized TELIP), which dissociates into the solution surrounding the liposome. (ii) Tightly bound exposed sctPA—integrally associated with the outer phospholipid bilayer of the liposome and does not dissociate under the physiological conditions (25–35 and 55–65% in the regular and optimized TELIP, respectively). The active (concave) and fibrin-binding sites of sctPA are exposed to the solution and functional. (iii) Tightly bound encapsulated sctPA—active sites are not exposed to the solution and are protected from high molecular weight (HMW) substrates or inhibitors (15–25 and 30–40% in the regular and optimized TELIP, respectively). Thus, in the optimized TELIP, almost 100% of active sctPA is tightly associated with the liposome. The infused octafluoropropane (OFP) gas is shown in green, and inactive sctPA is not shown.

Successful pharmacological treatment under conditions of slow fibrinolysis [1] requires the delivery of durable low-level plasminogen-activating activity to the site of injury. Liposomes loaded with tissue-type plasminogen activator (tPA) target clots through the fibrin-binding domains of tightly bound surface tPA, thereby potentiating thrombolysis [2,3,4,5,6,7,8]. The echogenic liposomal platform, ELIP, was initially proposed to enhance ultrasonographic imaging [9,10,11] and was later adapted for drug delivery and encapsulation [12,13,14], leading to the development of TELIP—single-chain (sc) tPA-loaded echogenic liposomal carriers with high affinity for fibrin and encapsulated enzymes [15,16]. Moreover, the encapsulated payload can be readily released from the ELIP by an ultrasound impulse [17,18]. TELIP was tested as a potential thrombolytic agent in a number of in vitro and in vivo translational studies [19,20,21]. In addition, a plasmin-loaded ELIP was prepared and characterized [7]. However, to the best of our knowledge, no FDA-approved systemically administered TELIP-based therapy has been reported to date. Encapsulating sctPA while simultaneously exposing its fibrin binding sites to the surrounding medium makes TELIP both a targeted carrier of the enzyme and a fibrinolysin [15]. Thus, TELIP is an attractive tool for the delivery of fibrinolytics [15,20,21,22,23], with the added capability of imaging ultrasound-based detection and activation. Ultrasound-mediated activation of TELIP accompanies local cavitation, which may affect fibrin structure and enhance the rate of thrombolysis [21,24]. Unfortunately, TELIP efficacy in preclinical circulation studies has been rather moderate [25], most likely due to rapid inhibition/clearance of sctPA in the bloodstream after injection and activation. Recent studies suggest that the addition of plasmin and magnetic nanoparticles, along with tPA and plasminogen in echogenic liposomes [7], increases the clot penetration and improves lytic efficacy [26,27,28]. However, plasmin/plasminogen in ELIP may require additional safety evaluation due to potentially increased risk of bleeding complications. A relatively high fraction of unbound/loosely bound sctPA, which was detected in the regular TELIP [19,22], may negatively affect lytic performance. Removing free sctPA from TELIP may improve the efficacy of thrombolytic/fibrinolytic therapy. We hypothesized (Scheme 1) that such optimization of the TELIP would enhance its unique properties when compared to the regular TELIP tested previously in translational research [19,20,21].

## 2. Materials and Methods

### 2.1. Proteins and Reagents

Human recombinant sctPA was obtained from Genentech (San Francisco, CA, USA). Human Glu-plasminogen and plasmin, thrombin, were obtained from Haematologic Technologies Inc. (HTI, Essex Junction, VT, USA). Human recombinant PAI-1, fibrinogen (Fbg) and FITC-fibrinogen (FITC-Fbg; 3 moles of fluorescein per mole of Fbg) were purchased through Molecular Innovations (Novi, MI, USA). Protein concentration was determined using a BCA protein assay kit (Pierce, Rockford, IL, USA). All experiments were carried out in physiological buffers: 20 mM Hepes/NaOH or 20 mM phosphate, pH 7.4, with 0.13–0.14 M NaCl, with or without bovine serum albumin (BSA;1.0 mg/mL).

### 2.2. Preparation of tPA-Loaded Echogenic Liposomes (Regular TELIP)

Echogenic liposomes were manufactured from four lipids: 1,2-distearoyl-sn-glycero-3-phosphocholine (DSPC), 1,2-dioleoyl-sn-glycero-3-phosphocholine (DOPC), 1,2-dipalmitoyl-sn-glycero-3-[phosphor-rac-1-glycerol] (DPPG), and cholesterol. All lipids were obtained from Avanti Polar Lipids (Alabaster, AL, USA) and were dissolved in chloroform at a molar ratio of 39:23:23:15. The solvent was evaporated under a stream of argon gas in a round-bottom flask rotated in a 50 °C water bath. Evaporation was completed per vacuo for at least 2 h. The lipid film was subsequently hydrated with Activase^®^, obtained from Genentech, Inc. (San Francisco, CA, USA), and reconstituted with nanopore water according to the manufacturer’s instructions (150 µg/150 µL per 5 mg lipid, followed by 350 µL 0.32 M D-mannitol per 5 mg lipid). The mixture was warmed to 42 °C while dispersing the lipids, followed by sonication for 5 min. Aliquots (5 mg lipid/0.5 mL) were distributed in microfuge tubes and frozen at −80 °C for at least 1 h, thawed at ambient temperature, and centrifuged at 13,200 rpm in a microfuge for 20 min. Pellets were resuspended in 0.5 mL of 0.32 M mannitol, transferred to glass vials, frozen at −80 °C for at least 2 h, and lyophilized in a Labconco FreeZone 4.5 L freeze dryer (Labconco Corporation, Kansas City, MO, USA). Dry cakes in sealed vials were stored at 4 °C until use. Lyophilized liposomes were gently reconstituted in water to a concentration of 10 mg/mL at room temperature for 5 min.

### 2.3. Single-Point tPA Plasminogen-Activating Activity Assay

The assay is a modification of the method of Ranby and Wallen [29], which assesses the ability of thrombolytics to convert plasminogen to plasmin, and was carried out in 96-well microtiter plates. Added to each well were 200 µL substrate-zymogen reagent (0.33 µM glu-plasminogen obtained from Millipore Sigma (Burlington, MA, USA) + 0.33 mM D-Val-Leu-Lys-*p*-nitroanilide obtained from Sigma-Aldrich (St. Louis, MO, USA) in 0.02 M phosphate-buffered saline (PBS), pH 7.4, with 0.145 M sodium chloride, followed by 20 µL of tPA standard (0.625–5.0 µg/mL) or TELIP (100–400X diluted) in PBS or PBS containing 0.1% Triton X-100 (PBS diluent). For the Triton X-100 treatment of TELIP, the reconstituted liposomes were first diluted 10X with 1.0% Triton X-100 and subsequently diluted further with PBS diluent. The wells were incubated for 10 min at room temperature, and 2 µL fibrin monomer solution (prepared as described for the Ranby–Wallen method) was added to each well and incubated for an additional 40 min at room temperature. The reaction was halted by adding 20 µL 4 M sodium acetate, at a pH of 3.8, to each well. Plates were read at 405 nm wavelength (A_405_) with a microplate reader.

### 2.4. Single-Point Solid-Phase Fibrin-tPA Activity Assay (SOFIA-tPA)

The assay was performed as previously described [15], with modification to measure PAI-1 inhibition of tPA activity. The first part of the assay is an enzyme immunoassay (EIA) protocol in which tPA (2 µg/mL PBS-T) and TELIP (100X diluted in PBS) are incubated for 2 h at 37 °C in fibrin-coated microplate wells. After washing with PBS containing 0.05% Tween-20 (PBS-T), wells were incubated with serial dilutions of PAI-1 (62.5–2000 ng/mL) for 1 h at 37 °C, followed by washing. The last assay incubation is the same as the tPA activity assay. The maximum percent activity inhibition for TELIP was compared to that for free tPA.

### 2.5. TELIP Characterization

TELIP echogenicity, size distribution and enumeration were assessed as previously described [30]. TELIP echogenicity was quantified from three replicate images generated using a 4V1c-S transducer probe (Siemens Medical Solutions USA, Inc. Mountain View, CA, USA) connected to a Siemens Acuson Sequoia 512 ultrasound system (Siemens Medical Solutions USA, Inc. Mountain View, CA, USA). Images were captured from video recordings of TELIP suspensions in an anechoic well over a RhoCee rubber pad (Precision Acoustics, Dorchester, UK) with an EZ Grabber Diamond VC500 unit situated between the PC and the ultrasound system that operated at a 2 MHz frequency and 0.2 MI. The mean gray scale values (MGSV; 0–255 scale) of captured images were determined with ImageJ software v1.54d. The echogenicity value was defined as the maximum MGSV of 400–12,800X dilutions (0.78–25.0 µg lipid/mL). Particle size characterization and enumeration of liposomes > 0.4 µm in diameter were performed with a Beckman Coulter Multisizer 4 instrument obtained from Beckman Coulter (Brea, CA, USA), using a 20 µm aperture tube. Size distribution analysis, including surface charge measurement of regular and optimized TELIP by dynamic light scattering (DLS), was performed using a Malvern Zetasizer instrument (Malvern Paralytical Ltd., Malvern, UK).

### 2.6. Continuous tPA Amidolytic and Plasminogen-Activating Activity Assays

Amidolytic and plasminogen-activating activities of tPA were measured using chromogenic substrate and clear 96-well flat-bottom plates, obtained from Corning Inc. (Austin, TX, USA), as previously described [31,32]. Briefly, samples of TELIP or control sctPA in 0.1 mL of 20 mM Hepes/NaOH buffer (pH 7.4) with 0.13 M NaCl and BSA (1.0 mg/mL) were mixed with an equal volume of 2.0 mM tPA chromogenic substrate. To measure plasminogen-activating activity, samples in 100 µL of the same buffer were mixed with plasmin chromogenic substrate and human Glu-plasminogen (final concentrations in 0.2 mL, 1.0 mM and 200 nM, respectively). Amidolytic and plasminogen-activating tPA activities were calculated from the initial slopes of changes in the A_405_ with time (t) and t^2^, respectively [31,32]. An enzyme with a known specific activity was used as a standard. A Synergy^TM^ HT Hybrid Reader, Cytation 5 and Cytation 10, obtained from Agilent Technologies (Santa Clara, CA, USA), were used to detect changes in Optical Absorbance at 405 nm with time as previously described [31,32,33].

### 2.7. Fibrinolytic Activity Assay

An in-house fibrinolytic assay [31,34,35] was used to measure fibrinolytic activity generated by TELIP in vitro, with or without PAI-1 added, as well as ex vivo. For ex vivo analysis TELIP (tPA loaded echogenic liposomes) was incubated with samples of pleural fluid collected prior to (baseline, 0 h) and after (final, 24 h) the treatment; 5 µL of a pleural fluid sample was mixed with 195 µL of 20 mM Hepes/NaOH buffer (pH 7.4; 0.13 M NaCl), with or without 5 nM sctPA, and was immediately transferred to a black 96-well flat-bottom plate with 20 µg of FITC-fibrin film at the bottom of each well. Fibrinolytic activity was calculated from the slopes of time-dependent increases in fluorescence intensity (excitation 490 nm, emission at 512 nm) due to the degradation of FITC-fibrin. An increase in the fluorescence emission at 512 nm with time due to de-quenching FITC-fluorescence was detected using a SynergyTM HT Hybrid Reader, Cytation 5 and Cytation 10 (Agilent Technologies, Santa Clara, CA, USA).

### 2.8. Comparison of Fibrinolytic Activity Generated by Regular TELIP and sctPA

TELIP or free sctPA at concentrations of 4.0, 2.0, 1.0 and 0.5 µg/mL were incubated in FPA96F wells in 50 µL of buffer (20 mM Hepes/NaOH, 0.14 M NaCl, 1 mg/mL BSA) for 2 h at 37 °C. After incubation, each well was washed three times with 100 µL of the same buffer. Following washing, 50 µL of either buffer or buffer containing PAI-1 (50, 25, 12.5, 6.25, 3.13, 1.56, 0.78 or 0.4 nM) was added into each well and incubated for 5 min. Subsequently, 50 µL of glu-plasminogen (200 nM) in the same buffer was added to each well. The reaction was monitored by recording changes in FITC fluorescence emission over time, and rates of fibrinolysis were calculated as previously described [32,33,36].

### 2.9. SDS PAGE Analyses

Total, active, encapsulated and inactive sctPA in TELIP samples were estimated from the results of the analysis of the products of the reaction with an excess of PAI-1 by SDS PAGE (4–12% gradient gel obtained from Invitrogen, Carlsbad, CA, USA), as previously described for tPA [36,37]. Briefly, samples of TELIP were preincubated with or without 0.1–0.5% Triton X-100 at room temperature in a physiological buffer (no BSA). A 2.0–4.0 molar excess of PAI-1 was added to the samples and incubated for another 5 min. The second set of samples was not treated with PAI-1. Samples of sctPA at the same concentrations were used as controls. SDS loading buffer (Invitrogen, Carlsbad, CA, USA) was added to each reaction mixture, heated at 100 °C for 2 min, and analyzed by SDS PAGE. The amounts of free sctPA (Mw = 63 KDa) and inhibitory complexes with PAI-1 (Mw = 110 KDa) were estimated from the intensity of the corresponding bands on gel scans using ImageJ shareware.

### 2.10. Effect of ELIP on the Stoichiometry of PAI-1 tPA Inhibition

Stoichiometry of inhibition (SI) is the number of molecules of PAI-1 required to inactivate one molecule of sctPA. The SI for the reaction between PAI-1 and tPA of TELIP was estimated from measurements of the residual tPA plasminogen-activating activity in TELIP samples, incubated with increased (up to 64-fold molar excess) of PAI-1 as previously described [36]. In short, 3–5 µM sctPA (in regular or optimized TELIP) was preincubated with or without 0.5% of Triton X-100 for 5 min at room temperature in 0.02 M Hepes/NaOH, pH 7.4, with 0.14 M NaCl. PAI-1 (0–64-fold molar excess) was added and incubated for 5 min. Aliquots were withdrawn for measurement of the residual tPA amidolytic and plasminogen-activating activity. The SI was calculated from 3 to 5 measurements and reported as mean ± standard error (S.E.). Free sctPA was used as a control. SDS loading buffer (Invitrogen, Carlsbad, CA, USA) was added to each reaction mixture. Samples were then heated at 100 °C for 2 min and analyzed by SDS PAGE. The amounts of PAI-1 complexed with tPA and free tPA not reacting with PAI-1 (encapsulated active and/or inactive) were estimated from the intensity of the corresponding bands using a BioRad Imaging system (Hercules, CA, USA), as described previously [32,33,38].

### 2.11. Preparation of Optimized TELIP and Evaluation of TELIP Integrity Under Physiological Conditions In Vitro

Lyophilized samples of regular TELIP (4 mg) were resuspended in 0.4 mL of water (10 mg/mL) at room temperature for 10 min and divided into four aliquots (100 µL each). Two aliquots (regular TELIP, aliquots 1 and 2, Ar1 and Ar2) were stored on ice, while the remaining two were centrifuged at room temperature (20 min, 14,000× *g*). The supernatants (S1) were collected, and the pellets were resuspended in 100 µL of the physiological buffer (0.02 M HEPES/NaOH, pH 7.3, 0.13 M NaCl) and centrifuged again. The supernatant (S2) was collected, and the resulting pellets (optimized TELIP, aliquots 1 and 2, Ao1 and Ao2) were resuspended again in the physiological buffer. A 5.8 µM sctPA solution was used as a control (C1). For sample preparation, the first set of 6 samples, including 20 µL of Ar1, Ao3 and C1, was mixed with 4 µL of 51 µM human PAI-1, and 20 µL of Ar1, Ao3 and C1 was mixed with 4 µL of the physiological buffer. For the second set, 40 µL of Ar1, Ao3 and C1 was first mixed with 1.0 µL of 20% Triton X-100. Subsequently, 20 µL of each sample was supplemented either with 4 µL of 51 µM PAI-1 or physiological buffer. Collected supernatants S1 and S2 were not treated with PAI-1. Each of the 16 samples (24 µL) was analyzed as follows: 5 µL of each sample was mixed with 195 µL of the physiological buffer that contained 1.0 mg/mL BSA per well of a 96-well clear plate and serially diluted 3 times. tPA amidolytic activity was measured in each sample using Pefachrome tPA substrate (Pentapharm AG, Aesch, Switzerland). The activity was calculated as the slope of an increase in the absorbance at 405 nm over time, and specific activity was estimated from the plots of activity vs. the concentration of tPA in the sample (C1) or expected concentration (Ar and Ao). A linear equation for activity, (AU) = b_0_ + b_1_[Enzyme], was fit to the data in order to determine specific activity in each sample. The rest of each sample (19 µL) was mixed with 6.0 µL of gel loading buffer, incubated at 100 °C for 2 min, and analyzed by SDS PAGE.

### 2.12. Evaluation of Optimized TELIP Integrity and Stability Under Physiological Conditions Ex Vivo

Optimized TELIP (2.5 mg/mL, 25 µg tPA/mg of lipid) was incubated either in 0.05 M HEPES/NaOH, pH 7.4 or in pleural fluid (PF) at 37 °C. Aliquots were withdrawn at 0–320 min, incubated with 6 μM human PAI-1 (with or without 0.5% Triton X-100 for 5 min), subjected to SDS PAGE, and stained with Gel Code Blue (in vitro samples) or with Western blot assayed for human tPA (ex vivo samples). SDS Mw standards were used to determine relative MW (KDa) and positions of PAI-1/tPA complex, free tPA, and excess PAI-1. Relative band densities (0 min = 100%) for PAI-1/tPA and free tPA with or without Triton X-100 were determined over 0–320 min and plotted against time to estimate the rates of spontaneous TELIP disintegration.

### 2.13. Effects of TELIP and sctPA on Fibrinolysis with Plasmin

A 96-well fibrinolytic activity assay [32,34,36,39] was used to determine the effect of TELIP (0–125 nM sctPA) and free sctPA (0–125 nM) on the rate of fibrinolysis with human plasmin (0–50 nM) of FITC-fibrin film (20 µg). The rate of fibrinolysis of FITC-fibrin was calculated from an increase in the fluorescence emission at 520 nm (excitation at 490 nm) over time. Controls (100%) represent the rates of fibrinolysis with plasmin. To evaluate the effects of fibrin-bound TELIP on fibrinolysis, FITC-fibrin films were incubated with 100 µL of 12.5 nM human plasmin alone (positive control), with plasmin and 200 nM sctPA (negative control), or with plasmin and optimized TELIP (equivalent of 200 nM sctPA) with or without exposure to an ultrasound pulse (20 kHz, 30% power, 1 s), using a Fisher Brand model 120 sonicator equipped with an eight-tip horn probe CL-18. Fibrinolytic activity was determined from an increase in the fluorescence emission at 520 nm (excitation at 490 nm) with time, as previously described [32,33,34].

### 2.14. Data Analysis and Statistics

Levels of statistical significance (α = 5%) were determined using the Kruskal–Wallis test and Dunn’s Multiple Comparison Test for column analyses of multiple groups. Likewise, the Mann–Whitney test was employed for column analyses of one or two groups. Statistical analyses and plots generated in this manuscript were performed using GraphPad Prism v10.6.0, as previously described [31,32,33,34,40].

## 3. Results

### 3.1. Production and Characterization of Regular TELIP

**Figure 1:**
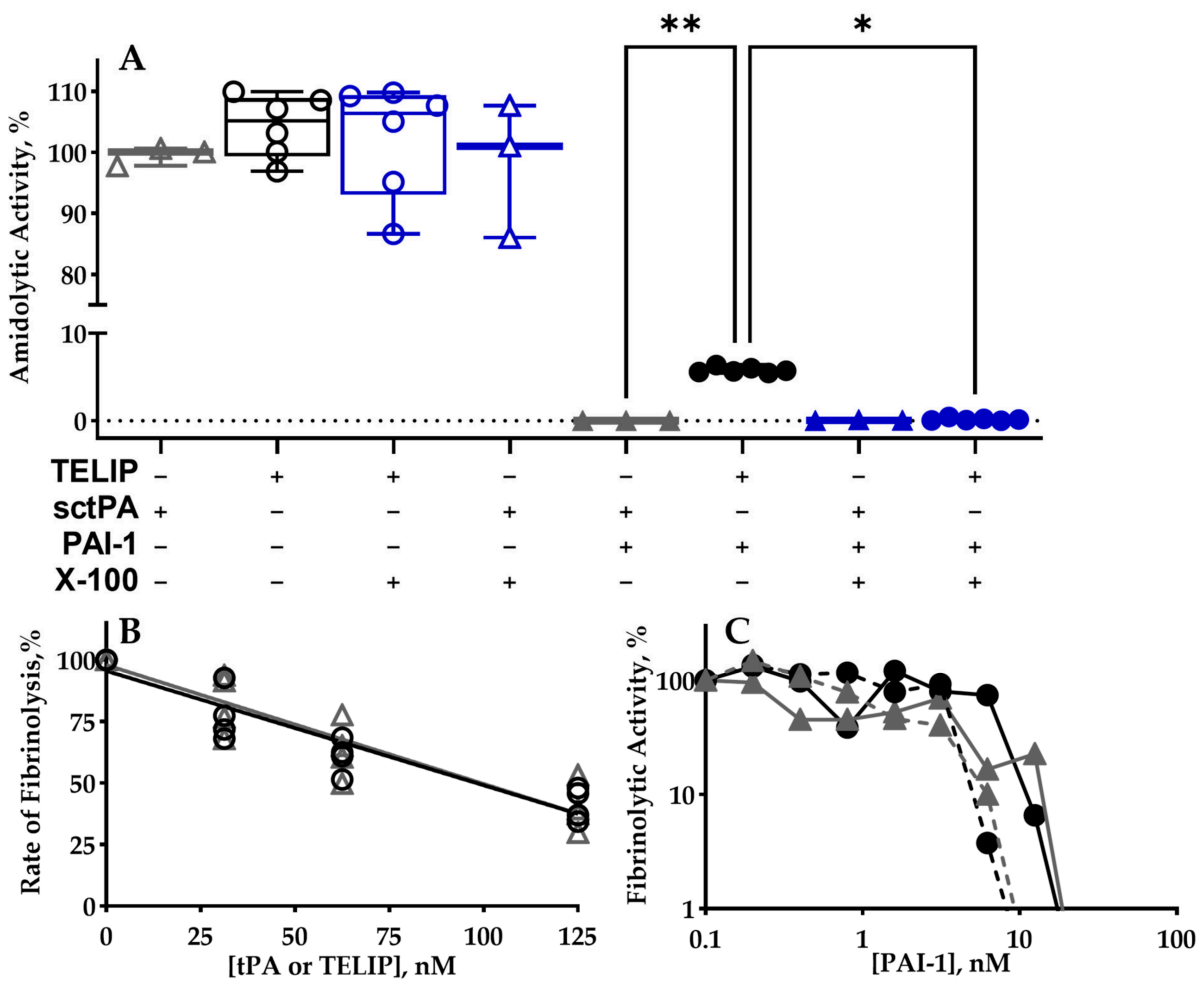
The regular TELIP contains a relatively small fraction of true encapsulated active sctPA and competes with plasmin for fibrin. (**A**) Amidolytic activity of samples of sctPA control (triangles) and regular TELIP (circles). From the left to right (per table placed under the plot): sctPA control (empty triangles; *n* = 3); regular TELIP (empty circles; *n* = 6); sctPA (*n* = 3) and regular TELIP (*n* = 6) in the presence of 0.1% Triton X-100 (blue symbols); same samples sctPA (*n* = 3); regular TELIP (*n* = 6), sctPA/0.1% Triton X-100 (*n* = 3); regular TELIP/0.1% Triton X-100 (*n* = 6) treated with 2-fold molar excess of PAI-1 (filled symbols). Approximately 6% of total amidolytic activity, which is resistant to PAI-1inhibition in a regular TELIP, reflects encapsulated active sctPA. Presence or absence of either TELIP, sctPA, PAI-1, X-100 in each group were presented as + or –, respectively. (**B**) Regular TELIP (empty circles, *d 3*) and sctPA (empty triangles, *n* = 3) affect fibrinolysis by plasmin. A 96-well fibrinolytic acidity assay [32,34,36,39] was used to determine the effect of TELIP (0–125 nM tPA) and sctPA (0–125 nM) on the rate of fibrinolysis with human plasmin (0–50 nM). The rate of fibrinolysis of FITC-fibrin was calculated from an increase in the fluorescence emission at 520 nm (excitation at 490 nm) with time. Controls (100%) represent the rates of fibrinolysis with plasmin. Regular TELIP and tPA decreased the rate of fibrinolysis, reflecting competition with plasmin for fibrin [41]. (**C**) Double logarithmic plot of effects of PAI-1 on fibrinolytic activity generated by 0.5 µg /mL (dashed lines) and 2.0 µg /mL (solid lines) of sctPA (triangles, *n* = 4) and regular TELIP (circles, *n* = 4). Statistical significance of groups with and without PAI-1 was determined using Kruskal–Wallis test and Dunn’s Multiple Comparison Test. A * denotes *p* < 0.05, and ** denotes *p* < 0.01.

Three batches of regular TELIP (I-III; Table 1) were produced, lyophilized, and characterized, as previously described [15,16]. The surface charge (~−80 mV) was unaffected by washing. Unlike tPA, which is inactivated by PAI-1 in stoichiometry close to unity, 5–15% of the amidolytic tPA activity in regular TELIP is protected from inactivation with an excess of PAI-1 (Figure 1A) and represents an enzyme that is encapsulated in the liposomal carrier (Scheme 1). Treatment with Triton X-100, which disintegrates the liposome structure, removes the protection of tPA from PAI-1 (Figure 1A). Notably, regular TELIP, similar to the free sctPA, competes with plasmin for fibrin, thus affecting the fibrinolytic activity of plasmin (Figure 1B). However, the large amount of free/loosely bound tPA in the regular TELIP (Scheme 1), which competes with plasmin, may affect the binding of liposomes to fibrin. No difference was observed between free tPA and regular TELIP in terms of the ability to support activation of plasminogen and fibrinolytic activity in the presence of increasing amounts of PAI-1 (Figure 1C). Thus, the tPA encapsulated in TELIP liposome (Scheme 1) is able to cleave low-molecular-weight (LMW) amidolytic substrate, but isolated from high-molecular-weight (HMW) inhibitor PAI-1 and substrate plasminogen. We hypothesized that free/loosely bound tPA in the regular TELIP (Scheme 1A) may affect liposomal carrier performance in vitro, ex vivo, and, potentially, in vivo.

### 3.2. Regular TELIP Contains 50–70% of Free/Loosely Bound Active sctPA, Which Dissociates from the Liposomes Under the Physiological Conditions

An optimized TELIP was produced by removing unbound/loosely bound sctPA from the regular TELIP. The free/loosely bound sctPA was removed by centrifugation of regular TELIP exposed to physiological pH and ionic strength, as described in the Materials and Methods section, and amidolytic activity in the samples was measured and compared to the original activity of the regular TELIP (Table 2).

Free/loosely bound sctPA was removed from the TELIP without affecting liposomes’ integrity and losses of the encapsulated active enzyme (resistant to inhibition with PAI-1; Table 2). The results of analyses of amidolytic activity in regular and optimized TELIP (Scheme 1) demonstrate a striking difference in fractions of tightly bound (43.2 and >90%) and true encapsulated sctPA (16.2 and >35%, respectively) (Table 2).

**Figure 2:**
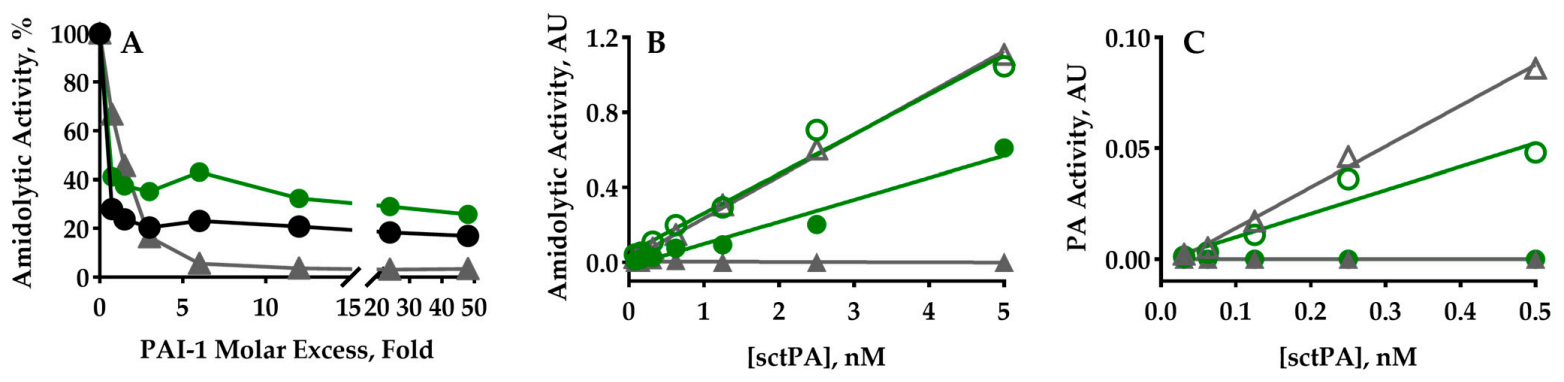
The optimized TELIP possesses a higher fraction of encapsulated sctPA than regular TELIP. (**A**) Changes in the amidolytic activity of the regular (black circles, *n* = 4) and optimized (green circles, *n* = 4) TELIP and sctPA (triangles, *n* = 4) with an increase in the molar fold of PAI-1. (**B**) Concentration dependence of amidolytic activity of optimized TELIP (green circles, *n* = 4) and matched control of sctPA (triangles, *n* = 4) with (filled symbols) or without (empty symbols) treatment with a 2-fold molar excess of PAI-1. (**C**) Plasminogen-activating activity of the same samples of optimized TELIP (green circles, *n* = 4) and sctPA (triangles, *n* = 4) with (filled symbols) or without (empty symbols) treatment with a 2-fold molar excess of PAI-1.

The optimized TELIP (Figure 2A) features a higher fraction of the encapsulated active sctPA. Unlike free sctPA, amidolytic activity of encapsulated enzyme (Scheme 1) was protected from complete inactivation with up to 50-fold molar excess of PAI-1 (Figure 2A). Stoichiometry of inhibition (SI, number of PAI-1 molecules needed to inactivate one molecule of the enzyme) close to unity was expected based on the molecular mechanism of the reaction between PAI-1 and tPA. Of note, similar values (1.7 ± 0.4, 1.4 ± 0.3 and 1.2 ± 0.2) of the SI were observed for the initial parts of the dependences for both regular and optimized TELIP and sctPA, respectively (Figure 2A). However, approximately 17–20% and 35–40% of the sctPA amidolytic activity in TELIP was resistant to up to 50-fold molar excess of PAI-1 (Figure 2A).

Comparison of the effect of PAI-1 on the samples of optimized TELIP and sctPA matched by amidolytic activity (Figure 2B) demonstrates that active sctPA was protected from inactivation with PAI-1. However, the plasminogen-activating activity of the same sample of optimized TELIP was lower than that of the sctPA control (Figure 2C), demonstrating isolation of the encapsulated sctPA from the HMW substrate plasminogen. Moreover, treatment with PAI-1 resulted in the complete inactivation of the plasminogen-activating activity in both samples, optimized TELIP and sctPA control (Figure 2C). Thus, the fraction of sctPA, tightly associated with the liposomes, is accessible by both HMW inhibitor (PAI-1) and substrate (plasminogen) if active sites are exposed to the solution (Scheme 1B). In contrast, encapsulated sctPA, which exhibits amidolitic activity towards LMW chromogenic substrate in the presence of up to 50-fold molar excess of PAI-1 (Figure 2A,B), expresses no plasminogen-activating activity (Figure 2C). Therefore, active sites of the encapsulated sctPA are likely shielded by the liposome from HMW ligands.

**Figure 3:**
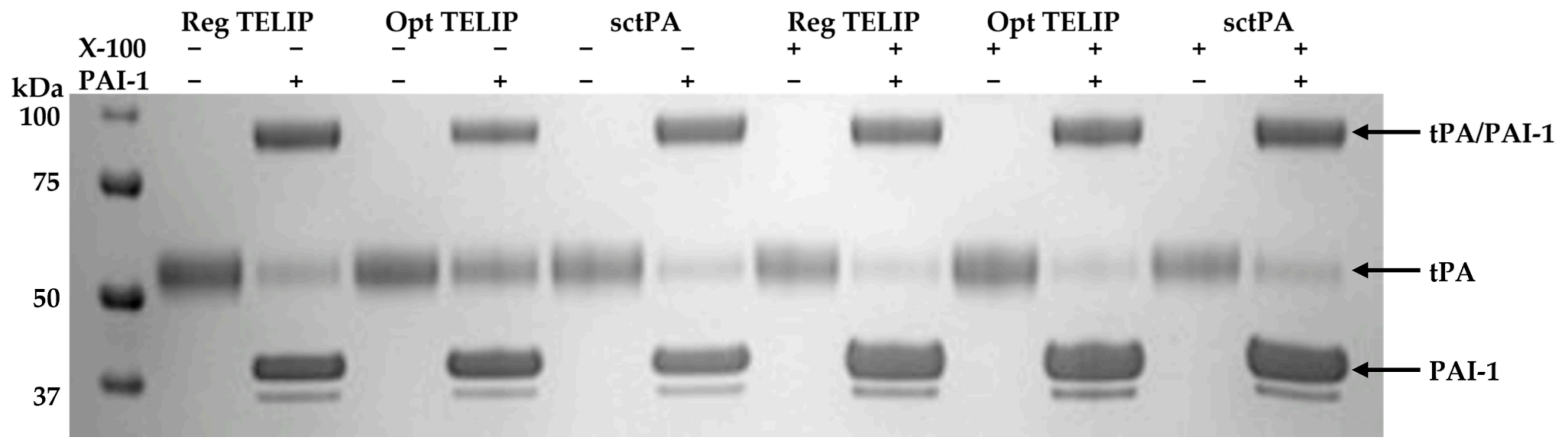
The optimized TELIP possesses a higher fraction of encapsulated sctPA, which is resistant to PAI-1, than the regular TELIP. SDS PAGE analyses of regular (Reg) and optimized (Opt) TELIP and sctPA (control) alone (−) or after (+) incubation with an excess of PAI-1, treated without (−) or with (+) Triton X-100, visualized for four species of sctPA: active encapsulated, tightly and loosely bound, and inactive.

The mechanism of protection of encapsulated sctPA was next verified with the SDS PAGE analysis of the regular and optimized TELIP (Table 2), sctPA control, and products of their reactions with 2-fold molar excess of PAI-1, with and without the presence of Triton X-100, which lyses liposomes and releases both tightly bound and encapsulated enzyme into the solution (Figure 3).

The results of SDS PAGE analysis (Figure 3) support conclusions derived from the activity measurements (Table 2, Figure 2). The relative amount of encapsulated sctPA (resistant to the reaction with PAI-1 without Triton X-100) in the optimized TELIP is 2-fold higher than that in the regular TELIP. SDS PAGE analysis visualizes the relative amounts of the enzymatically inactive sctPA (does not react with PAI-1 in the presence of Triton X-100) in regular and optimized TELIP. Since fractions of the enzymatically inactive sctPA in all preparations were similar (Figure 3, lanes 1, 3, and 5 from the right), the origin of the inactive sctPA is probably an enzyme that was used for the production of the regular TELIP (Table 1). Next, samples of the optimized TELIP were evaluated for their ability to induce, support and affect fibrinolysis.

### 3.3. Enhancement of the Unique Features of the Echogenic Liposomal Carriers in the Optimized TELIP

**Figure 4:**
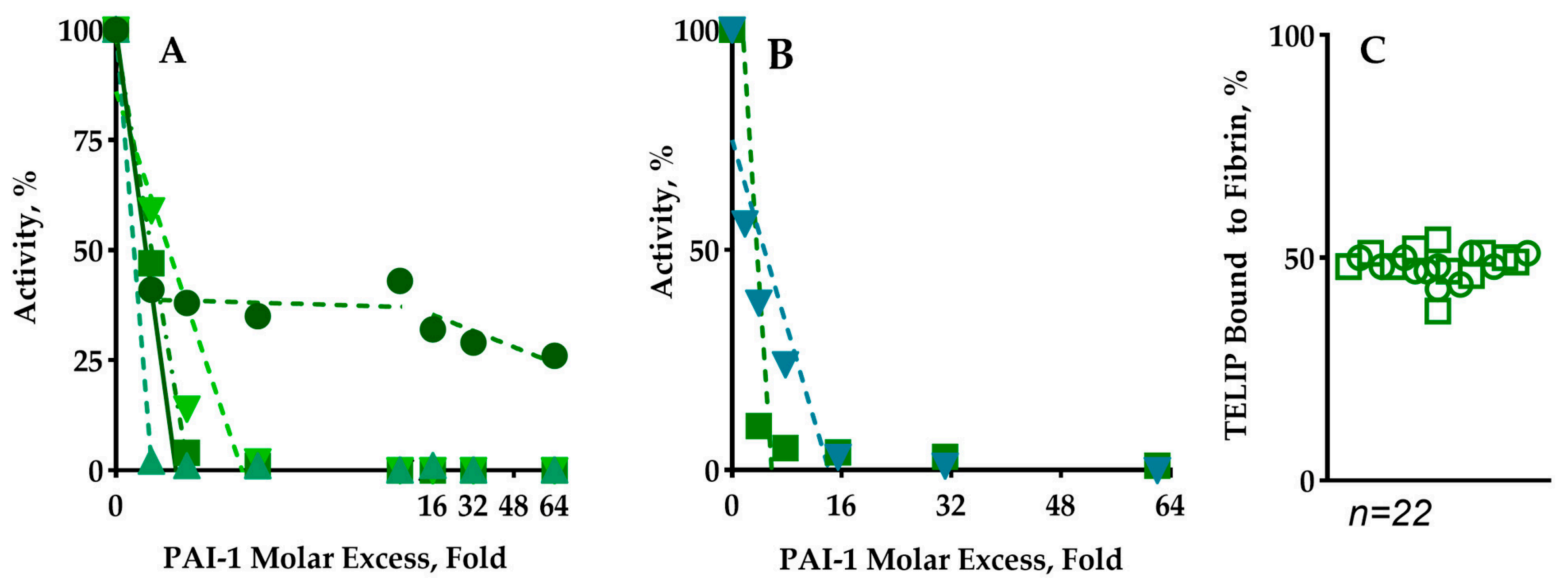
The optimized TELIP binds to fibrin with high affinity and induces fibrinolysis in the presence of up to 15X molar excess of PAI-1. (**A**) Optimized TELIP (% of 200 ng) bound to fibrin (empty diamonds, *n* = 11) or FITC-fibrin (empty squares, *n* = 11) films (20 µg) at the bottom of a well in a 96-well plate. (**B**) Dependence of plasminogen-activating (**B**) and fibrinolytic (**C**) activities of the optimized TELIP (green circles, *n* = 4) on molar excess of PAI-1 (filled symbols, *n* = 8 in two repeats) added in buffer solution with (solid green lines) or without fibrin (dashed green lines).

Up to 100 ng of the optimized TELIP binds to fibrin or FITC-fibrin film (20 µg) with high affinity (Figure 4A). To determine the effect of fibrin on the activity and reaction with PAI-1, the optimized TELIP bound to fibrin or FITC-fibrin film was exposed to increasing amounts (i.e., up to 64-fold molar excess) of PAI-1, and residual plasminogen-activating (Figure 4B) and fibrinolytic (Figure 4C) activities were measured as previously described [36].

**Figure 5:**
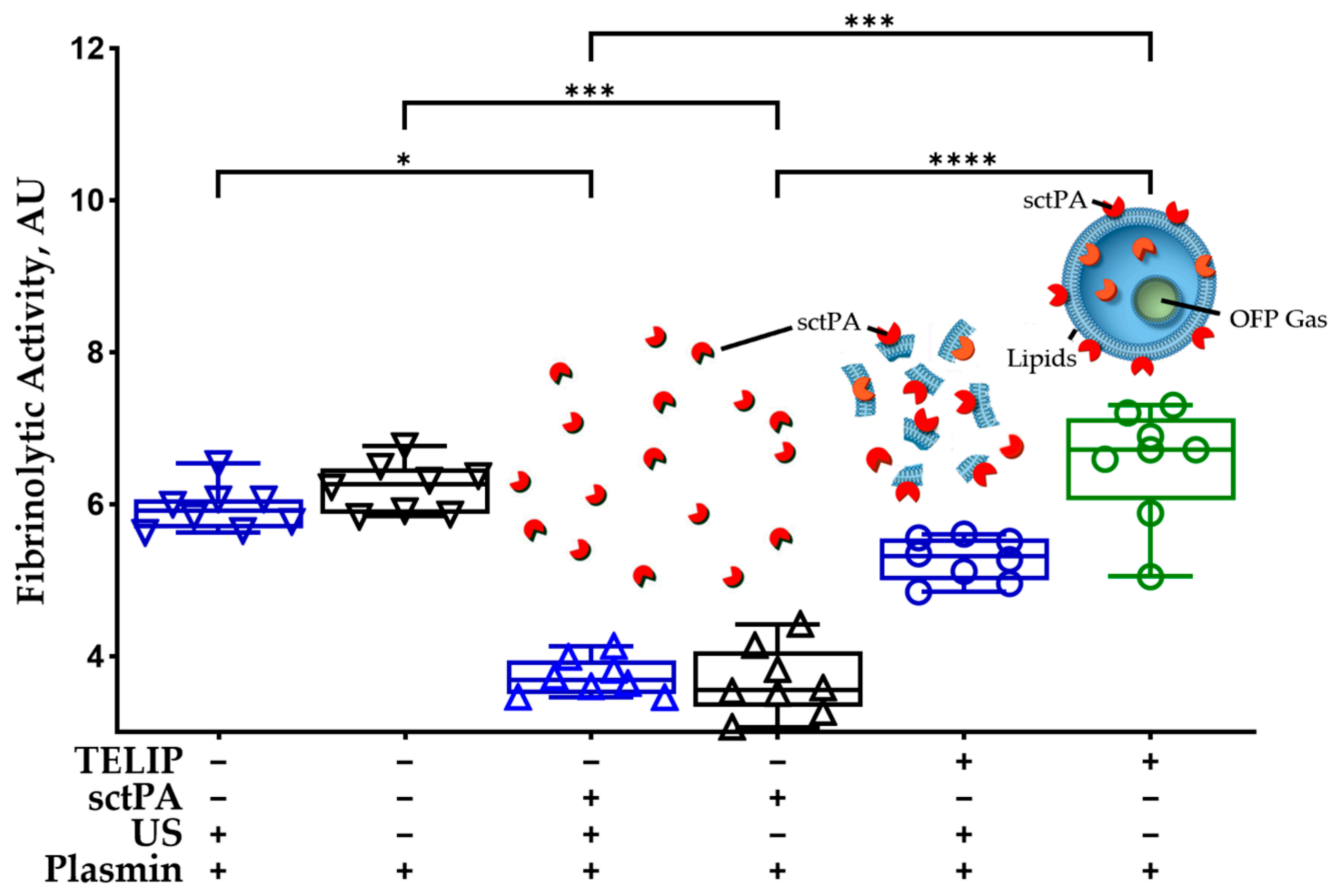
Optimized TELIP bound to fibrin does not interfere with fibrinolysis induced by plasmin. Each well of a 96-well plate (*n* = 8 each) contained 100 µL of 12.5 nM human plasmin incubated with a FITC-fibrin film (20 µg) with and without a single pulse of ultrasound (US; Fisher Brand Model 120, 30% power, 1 s) treatment. The table below and illustrations above the boxes represent composition of the reaction mixtures: (from left to right) plasmin with (blue reverse triangles, *n* = 8) and without (−, black reverse triangles, *n* = 8) ultrasound exposure; plasmin and 200 nM free sctPA with (blue triangles, *n* = 8) and without (black triangles, *n* = 8) ultrasound exposure; plasmin and optimized TELIP (equivalent of 200 nM sctPA) exposed to ultrasound pulse (blue circles, *n* = 8); plasmin and intact optimized TELIP (equivalent of 200 nM sctPA, green circles, *n* = 8). The presence or absence of TELIP, sctPA, US, plasmin in the x-axis were represented as + or −, respectively. Fibrinolytic activity was estimated from an increase in the fluorescence emission at 520 nm (excitation at 490 nm) with time, as previously described [32,33,34]. Statistical significance of data was determined by using Kruskal–Wallis test and Dunn’s Multiple Comparisons test. A * denotes *p* < 0.05, *** denotes *p* < 0.001, and **** denotes *p* < 0.0001.

Next, we studied the effects of the fibrin-targeted delivery and release of encapsulated sctPA with an ultrasound pulse on fibrinolysis. First, the effects of the optimized TELIP on the rate of fibrinolysis with human plasmin (Figure 5) were compared to that of free tPA using our fibrinolytic activity assay [32,33,34]. While the rate of fibrinolysis of FITC-fibrin with human plasmin decreased in the presence of tPA (*p* < 0.0001), optimized TELIP caused minimal (if any) effect (*p* > 0.05). However, after exposure of the optimized TELIP to the ultrasound pulses, the rate of fibrinolysis decreased (*p* < 0.0001), probably due to the release of encapsulated sctPA, which competes with plasmin for fibrin (Figure 5). Thus, optimized TELIP successfully delivers and releases encapsulated sctPA to the target, without a significant effect on the rate of fibrinolysis.

**Figure 6:**
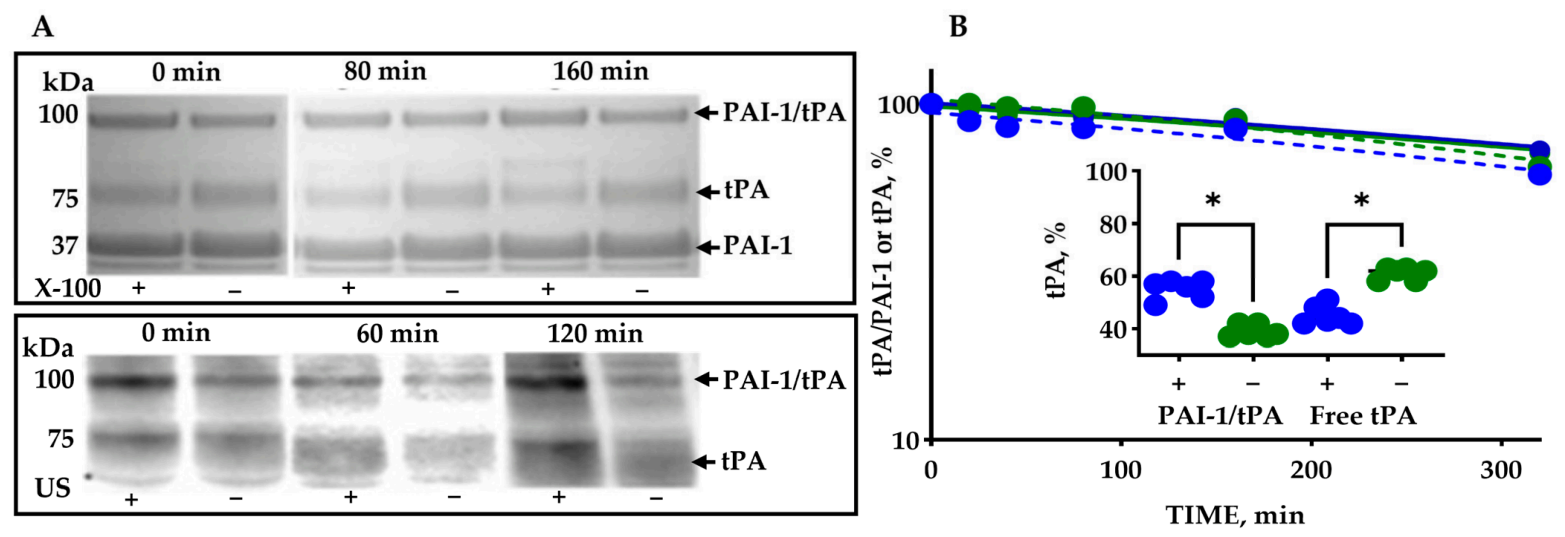
Stability of the optimized TELIP in vitro and ex vivo. (**A**) Optimized TELIP (2.5 mg/mL, 25 µg tPA/mg of lipid) was incubated at 37 °C either in 0.05 M HEPES/NaOH, pH 7.4 (top panel) or in pleural fluid (bottom panel). Samples were withdrawn, incubated with 6 μM human PAI-1 with (+) or without (−) 0.5% Triton X-100 for 5 min (top panel) or exposed (+) or not (−) to an ultrasound (US) pulse (Fisher Brand Model 120, 30% power, 1 s), subjected to SDS PAGE and stained with Gel Code Blue (top panel) or with Western blot assayed for human tPA (bottom panel). Samples shown are collected at 0, 80, 160 min and 0, 60, 120 min for buffer and pleural fluid incubations, respectively. Relative MW (KDa) and positions of PAI-1/tPA complex, free tPA and excess of PAI-1, respectively, are shown at the sides (black arrows). (**B**) A semilogarithmic plot of changes in the relative (0 min = 100%) band density for PAI-1/tPA (solid lines, *n* = 6) and free tPA (dashed lines, *n* = 6) with (blue circles) or without (green circles) Triton X-100, respectively. Lines represent the best fit of a linear equation to each set of data with slopes of 0.94, 1.26, 0.97 and 1.35 × 10^−3^ min^−1^, respectively. Inset: relative distribution of free and complexed with PAI-1 tPA band density (D_100%_ = D_free tPA_ + 0.6D_PAI-1/tPA_) in samples treated with PAI-1 with (+, blue circles, *n* = 6) or without (−, green circles, *n* = 6) 0.5% Triton X-100. Samples were withdrawn at 0, 20, 40, 80, 160 and 320 min. Statistical significance of data was determined using the Mann–Whitney test with a * denoting *p* < 0.05.

The stability and integrity of the optimized TELIP at 37 °C in vitro and ex vivo were evaluated for up to five hours (Figure 6). The time interval (0–5 h) was selected to include the projected time of ultrasound-mediated release of the encapsulated sctPA for the circulation (<1 h) and for slow intrapleural fibrinolysis (2–4 h). The optimized TELIP was incubated at 37°C in the buffer solution (Figure 6A; upper panel) or in the presence of the pleural fluid collected from a model of empyema in rabbits (Figure 6A; bottom panel). While the liposomes in the buffer solution were lysed with 0.5% Triton X-100, those incubated with the pleural fluid were treated with an ultrasound pulse (30% power, 1 s). The changes in tPA activity and encapsulated sctPA in the samples incubated in the buffer over time are shown in Figure 6B. Fractions of tPA, free and complexed with PAI-1 (as determined by densitometry), decrease and increase, respectively, after treatment with 0.5% Triton X-100 (Figure 6B; Inset, blue), reflecting the presence of the encapsulated sctPA.

Optimized TELIP has demonstrated stability and integrity in vitro and ex vivo, retaining >80% of total and >90% of encapsulated tPA activity, after incubation for 5 h in a physiological buffer (Figure 6B) or in the empyema pleural fluid at 37 °C. The half time of inactivation of external and encapsulated tPA at 37 °C was in the range of 9–11 h (Figure 6B), with no significant changes noted in distribution of the enzyme between two species of sctPA in the optimized TELIP: (i) tightly bound to the liposome and exposed to the solution and (ii) encapsulated and protected from the HMW inhibitors and substrates during up to 5.3 h incubation under the physiological conditions (Figure 6).

## 4. Discussion

The goal of the present study was an in vitro and ex vivo comparison of the regular and optimized TELIP in order to evaluate potential advantages of the latter for preclinical drug development studies and to test optimized TELIP in a model of acute empyema in rabbits [31,32,33,34,40]. First, we developed a comprehensive approach to TELIP characterization, which enables the assessment of sctPA distribution among four species: loosely bound, tightly bound, encapsulated active sctPA, and inactive enzyme. Since the specific plasminogen-activating activity of tPA may change due to its activation by fibrin [42,43], SDS PAGE (Figure 3) and amidolytic activity (Figure 1 and Figure 2) of the products of the reaction with PAI-1 allow for the visualization of all four species of sctPA in both the regular and optimized TELIP. Changes in the plasminogen-activating and amidolytic activity in the presence of PAI-1 clearly indicate inhibition of sctPA, which is loosely/tightly bound to the surface of the liposome (Figure 1A, Figure 2, Figure 3, Figure 4B and Figure 6). In contrast, the enzyme that is encapsulated in the TELIP lumen (Scheme 1B) and possess amidolytic activity towards LMW substrate is resistant to the multifold molar excess of PAI-1 (Figure 2, Figure 3 and Figure 6). The mechanism of protection of the encapsulated sctPA activity from PAI-1 differs from those known for PAI-1-neutralizing monoclonal antibodies [37,44,45,46]. Thus, PAI-1 readily inactivates all the sctPA in the solution and on the surface of the liposome but is unable to interact with the encapsulated enzyme (Figure 1A, Figure 2, Figure 3, Figure 4 and Figure 6) due to the restriction of diffusion into the lumen of the liposome for HMW proteins. Retaining amidolytic activity in the presence of PAI-1 by TELIP recapitulates α-macroglobulin/urokinase “molecular cage”-type complexes, which may slowly degrade in vivo, supporting fibrinolysis with low-grade plasminogen-activating activity [40,47]. In contrast, the majority of the encapsulated plasminogen-activating activity of TELIP delivered to the site of the injury (thrombus/fibrin clot) may be fast released in a timely manner with a short exposure to the ultrasound (Figure 5 and Figure 6).

Up to 60% of total enzymatic activity in regular TELIP dissociates under physiological conditions (pH and ionic strength) and is detected in the supernatants after centrifugation of the liposome suspension (Table 2). It appears that non-specific binding of sctPA to the liposome surface is most likely due to electrostatic interactions. Thus, under physiological conditions, regular TELIP represents a mixture of free sctPA and optimized TELIP (Scheme 1). Free sctPA with high affinity for fibrin may outcompete both liposomes and plasmin (Figure 1B and Figure 5), thereby affecting both targeted delivery with TELIP and the rate of thrombolysis/fibrinolysis mediated by plasmin. Since binding to fibrin protects plasmin from rapid inactivation with α_2_-antiplasmin [48], competition with sctPA in vivo may also promote loss of active plasmin. These potentially adverse effects of loosely bound sctPA on the efficacy of the regular TELIP may have contributed to the equivocal results seen in a previous study [20].

Unlike regular TELIP, optimized TELIP contains minimal amounts (<10–15%) of loosely bound sctPA and its enzymatic activity is distributed between those encapsulated (30–40%) and those exposed to the solution tightly bound (50–60%) sctPA molecules, with a maximal tPA load of 25–30 µg/mg lipid (Scheme 1B). Optimized TELIP exhibits almost no competition with plasmin (Figure 5) and affects fibrinolysis only after the fragmentation of the liposome and release of the encapsulated enzyme with ultrasound. Minimal competition between plasmin and optimized TELIP (Figure 5) likely originates from an at least two orders of magnitude smaller size of the enzyme molecule, and a limited number of liposomes bound to the fibrin surface (Figure 4A). The plasminogen-activating activity of sctPA tightly bound to the surface of the liposome, when delivered to the fibrin surface, may support thrombolysis/fibrinolysis at the site of injury with minimal systemic effects. Notably, binding to fibrin protects sctPA associated with liposomes from inactivation with endogenous PAI-1 (Figure 4B), likely by redirecting the reaction towards the substrate branch due to stabilizing the transient acyl-enzyme intermediate. Furthermore, the release of the TELIP payload by ultrasound may also induce local cavitation [21,24] and disrupt the structure of the thrombus/fibrin clot, thereby further increasing the efficacy of thrombolysis/fibrinolysis. Interestingly, the sctPA load for the regular TELIP was reported to be as high as 30–40 µg/mg [19,21,49], with more than 90% activity sensitive to PAI-1 [19], which may reflect a variation in the fraction of free/loosely bound sctPA in the preparations. In contrast, liposomes purified from different preparations of the original TELIP possess relatively stable fractions of tightly bound and encapsulated sctPA. Thus, large amounts of free sctPA in the regular TELIP could mask the clear benefits of the ELIP platform, designed to support rapid fibrin degradation and targeted delivery of the plasminogen activator. As expected, the negative control—ELIP liposomes without sctPA load—possessed no enzymatic activity or high affinity to the target when compared with either regular or optimized TELIP.

In summary, optimized TELIP possesses unique features that make it an attractive adjunct for targeted delivery of plasminogen-activating activity to fibrin with minimal adverse effects on the rate of fibrinolysis. Optimized TELIP lacks loosely bound sctPA (Scheme 1), which may compete with both liposomes and plasmin for the target (fibrin) with regular TELIP (Figure 1B and Figure 5). Optimized TELIP effectively binds to fibrin and does not affect the plasmin-mediated fibrinolysis (Figure 4A and Figure 5). Binding to fibrin protects enzymes exposed to the solution (50–60% of total activity) from inactivation with PAI-1 (Figure 4B), increasing the half-life of the plasminogen-activating activity and delivering to the target up to 40% of the encapsulated enzyme (Figure 2, Figure 3 and Figure 6). Optimized TELIP is stable and retains its structural integrity up to 5 h in vitro and ex vivo (Figure 6), and encapsulated sctPA may be released in a timely manner at the site of the injury by an ultrasound impulse (Figure 5 and Figure 6A, bottom panel). Finally, ultrasound-mediated cavitation [21,24] may affect fibrin structure, thus enhancing the fibrinolysis. Future preclinical studies are needed to evaluate optimized TELIP to determine the relative contribution of these features to the treatment efficacy.

## 5. Conclusions

Optimized TELIP possesses a number of unique features that make it a promising candidate for preclinical testing in validated animal models.

## Figures and Tables

**Table 1:** Production and characterization of regular (Scheme 1A) TELIP.

Batch No.	Echogenicity (Max. MGV)	Liposome Diameter (nm)	Liposome No./mg Lipid (×10^−9^)	Loading Efficiency (Activity)			
				Total Liposome		Washed Pellet	
				µg/mg Lipid	%	µg/Mg Lipid	%
I	97.1	676	3.98	29.2	97.5	29.2	97.5
II	93.9	930	3.24	29.6	98.5	25.6	85.4
III	107.8	687	2.11	31.6	100	24.6	82.0
Mean ± SD	99.6 ± 7.3	764 ± 144	3.11 ± 0.94	30.1 ± 1.3	98.7 ± 1.3	26.5 ± 2.4	88.3 ± 8.2

**Table 2:** Active sctPA (>50%) in the regular TELIP is loosely bound to liposomes and dissociates under physiological conditions. After exposure of the regular TELIP to physiological pH and ionic strength, unbound sctPA was separated by centrifugation (supernatant 1), and pellets were washed (supernatant 2), yielding the optimized TELIP.

	Buffer		Buffer/Triton ×100	
	No PAI-1	2× PAI-1	No PAI-1	2× PAI-1
Regular TELIP *	100.0 ± 13.0	16.2 ± 2.0	100.0 ± 10.0	3.4 ± 1.0
Optimized TELIP	43.2 ± 4.0	16.9 ± 2.0	57.0 ± 6.0	3.3 ± 1.1
Supernatant 1	22.8 ± 3.0	ND	32.0 ± 4.0	ND
Supernatant 2	31.6 ± 4.0	ND	21.0 ± 3.0	ND
sctPA, control	100.0 ± 11.0	0.60 ± 1.5	82.6 ± 12.0	1.4 ± 0.8

* Amidolytic activity is shown as a percentile of that for the regular (Scheme 1A) TELIP (100%) for all samples excluding sctPA controls; ND—not determined.

## Data Availability

The original contributions presented in this study are included in the article. Further inquiries can be directed to the corresponding author.

## Abbreviations

The following abbreviations are used in this manuscript:

BSA	bovine serum albumin
ELIP	echogenic liposomes
FITC	fluorescein isothiocyanate label
HMW	high molecular weight
LMW	low molecular weight
MI	mechanical index
OFP	Octofluoropropane
PAI-1	plasminogen activator inhibitor
sctPA	single-chain tissue-type plasminogen activator
SI	stoichiometry of inhibition
SDS PAGE	sodium dodecyl sulfate polyacrylamide gel electrophoresis
TELIP	sctPA-loaded ELIP
